# Erratum

**DOI:** 10.1111/j.1582-4934.2008.00393.x

**Published:** 2008-08-11

In [1], the footnote symbol (^†^) noting the two authors who contributed equally to the work was incorrectly attributed to C. Habold and R. Schneider-Stock. However, the two authors who in fact contributed equally to the work were C. Habold and A. Poehlmann.

In [2], the author affiliation (^a^) was incomplete. The correct affiliation is as follows:

Vaccine and Infectious Disease Institute (VIDI), Laboratory of Experimental Hematology, Faculty of Medicine, Antwerp University, Belgium.

We apologize for these errors.

## References

[1] Habold C, Poehlmann A, Bajbouj K, Hartig R, Korkmaz KS, Roessner A, Schneider-Stock R (2008). Trichostatin A caused p53 to switch oxidative-damaged colo-rectal cancer cells from cell cycle arrest into apoptosis. J Cell Mol Med.

[2] Cools N, Van Tendeloo VFI, Smits ELJM, Lenjou M, Nijs G, Van Bockstaele DR, Berneman ZN, Ponsaerts P (2008). Immunosuppression induced by immature dendritic cells is mediated by TGF-β/IL-10 double-positive CD4^+^ regulatory T cells. J Cell Mol Med.

